# Supplementary material for: Creative destruction: Sparse activity emerges on the mammal connectome under a simulated communication strategy with collisions and redundancy
Source: Netw Neurosci. 2020 Nov 1;4(4):1055–71. doi: 10.1162/netn_a_00165 (PMC7655042; doi:10.1162/netn_a_00165)
Supplement: Supplementary file 1 [file netn-04-1055-s001.pdf]

Hao, Y., & Graham, D. (2020). Supporting information for "Creative destruction: Sparse activity emerges on the mammal connectome under a simulated communication strategy with collisions and redundancy." *Network Neuroscience*, *4*(4), 1055-1071. https://doi.org/10.1162/netn_a_00165

Supplemental Material

Creative destruction: Sparse activity emerges on the mammal connectome under a simulated communication strategy with collisions and redundancy.

Yan Hao and Daniel Graham

A

Algorithm 1 Information Spreading

```
procedure MESSAGE FEED
    N ← the number of states in the network
    Adj ← the adjacency matrix of the networks
    n_batch ← how many batches of messages will be fed to the network
    load ← number of messages in each batch
    Msg ← load random sender and receiver node pairs
    Senders ← all sender nodes in Msg
    Activity ← n_batch × N matrix of 0
    Activity(1, Senders) ← 1
    Attempts(1, Senders) ← 1
end procedure
procedure MESSAGE DISTRIBUTION
loop:
    for i ← 2, n_batch do
        Activity(i, :) ← Activity(i − 1, :) × Adj
        Msg ← load random sender and receiver node pairs
        Senders ← all sender nodes in Msg
        NewActivity ← 1 × N vector of 0
        NewActivity(Senders) ← 1
        Activity(i, :) ← Activity(i, :) + NewActivity
        Attempts(i, :) ← Activity(i, :)
        Where ← where entries of Activity(i, :) are ≥ 1
        Activity(i, Where) ← 0
        Attempts(i, Where) ← 1
    end for
end procedure
```

B

Algorithm 2 RandomWalk

```
procedure MESSAGE FEED
    n_batch ← how many batches of messages will be fed to the network
    load ← number of messages in each batch
    Msg ← load random sender and receiver node pairs
    Activity(1, :) ← all sender nodes in Msg
    Attempts(1, :) ← all sender nodes in Msg
end procedure
procedure MESSAGE DISTRIBUTION
loop:
    for i ← 2, n_batch do
        for j ← 1, length of Activity(i − 1, :) do
            PossibNext ← all nodes reachable from Activity(i − 1, j)
            NextMsg(j) ← a randsample from PossibNext
        end for
        Msg ← load random sender and receiver node pairs
        Activity(i, :) ← [NextMsg, Msg]
        Attempts(i, :) ← all unique entries in Activity(i, :)
        Jams ← all repeated elements in Activity(i, :)
        if Jams ≠ ∅ then
            for k = 1, length of Jams do
                Where ← positions of entries in Activity that equal to Jams(k)
                Activity(i, Where) ← 0
            end for
        end if
    end for
end procedure
```

Box S1. (A) Pseudocode of information spreading (IS) model. (B) Pseudocode of random walk (RW) model.

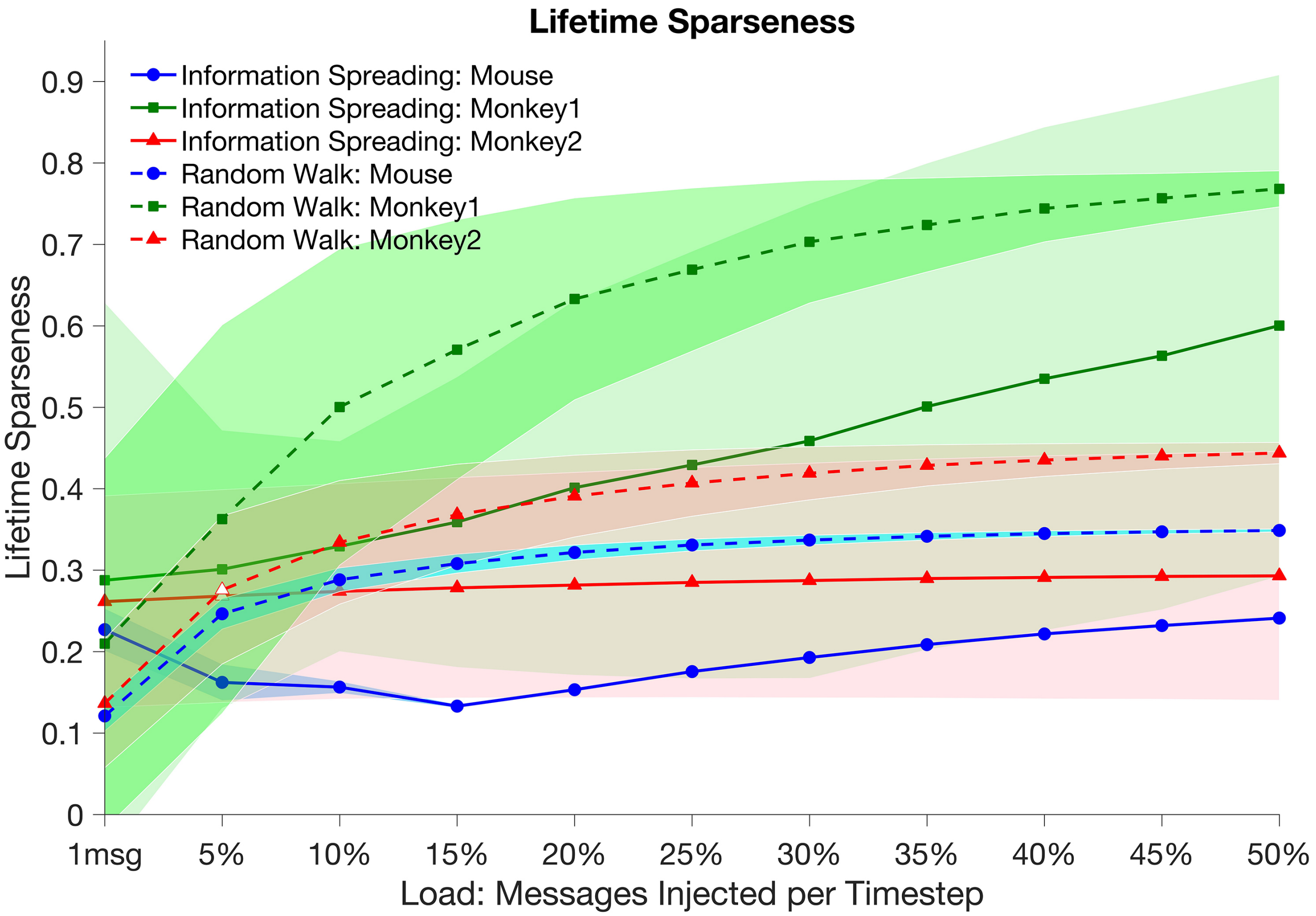

Figure S1. Mean lifetime sparseness of IS (solid lines) and RW (dashed lines) models as a function of load. As with population sparseness, lifetime sparseness is greater (closer to 0) for IS models compared to RW at loads above 1 message per time step. Shaded areas indicate 2 standard deviations from the mean. Filled symbols indicate significant differences (t-test, p<0.01) between corresponding IS and RW data, whereas open symbol indicates no significant difference.

A

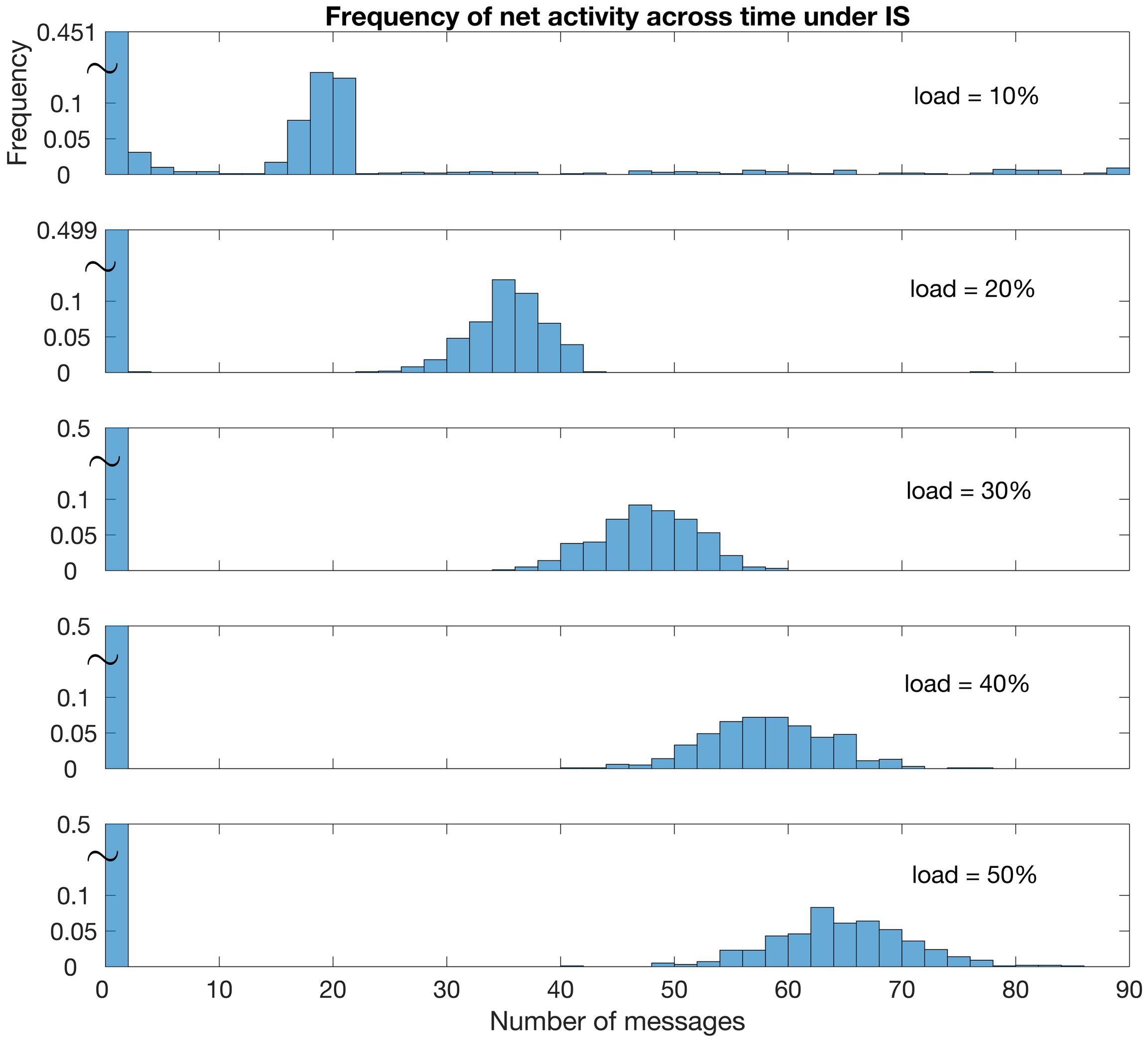

Frequency of net activity across time under IS
Frequency
0.451
0.1
0.05
0
load = 10%
0.499
0.1
0.05
0
load = 20%
0.5
0.1
0.05
0
load = 30%
0.5
0.1
0.05
0
load = 40%
0.5
0.1
0.05
0
load = 50%
0
10
20
30
40
50
60
70
80
90
Number of messages

B

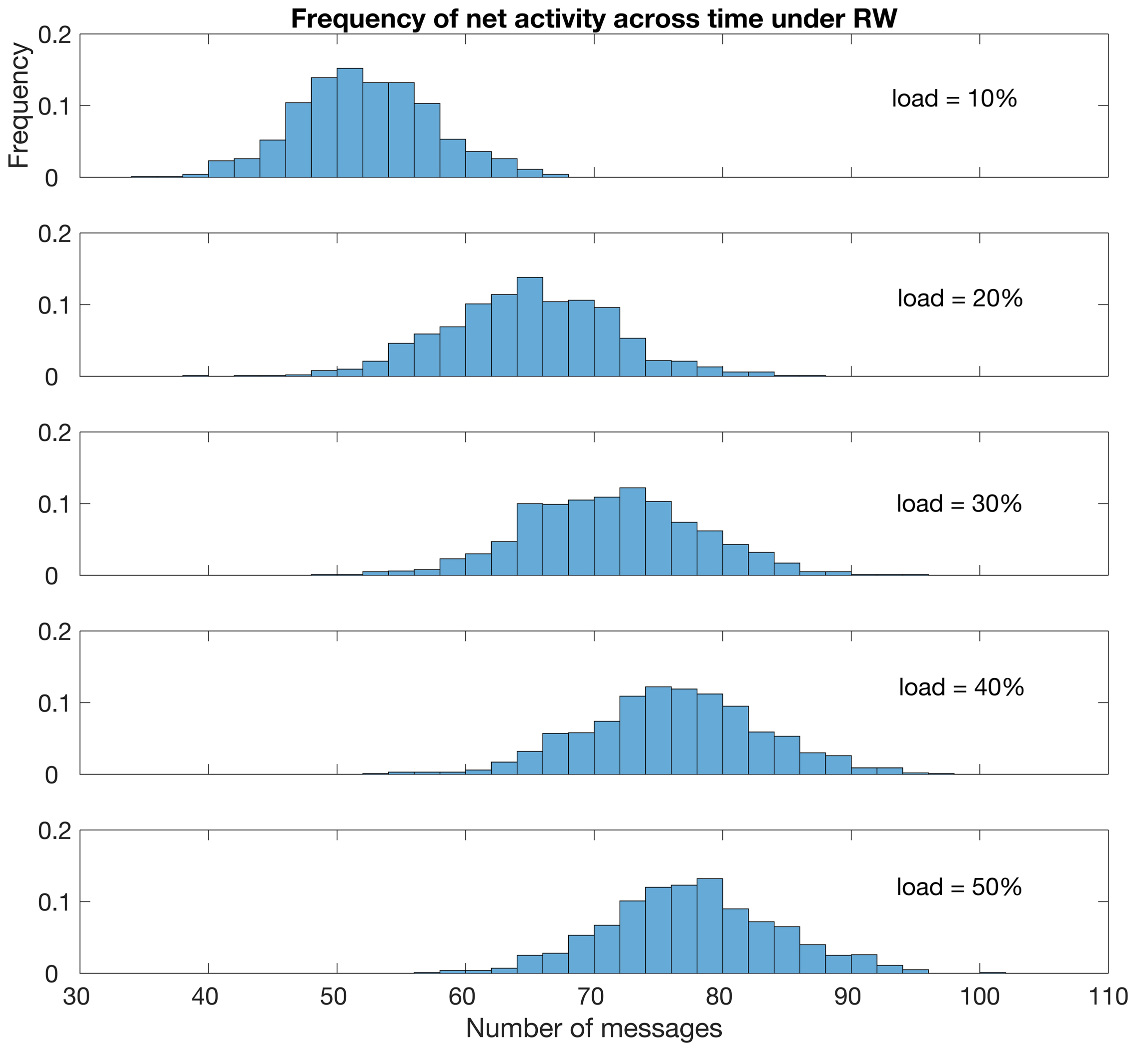

Figure S2. Relative frequency of net activity under IS models (A) and under RW models (B) at different loads. Results are for the *mouse* network**.** Tildes in A indicate a contracted vertical axis.
